# Prevalence and Factors Associated with Impaired Maternal–Infant Bonding among Mothers Attending Young Child Clinic in Kampala, Uganda

**DOI:** 10.3390/ijerph21060665

**Published:** 2024-05-23

**Authors:** Phionah Tukamushabe, Tom Denis Ngabirano, Joyce Nankumbi Okonya, Melissa A. Saftner

**Affiliations:** 1 Department of Nursing, Makerere University, Kampala P.O. Box 7072, Uganda; ptukamushabe@jcrc.org.ug (P.T.); tom.ngabirano@mak.ac.ug (T.D.N.); joyce.nankumbi@mak.ac.ug (J.N.O.); 2 School of Nursing, University of Minnesota, Minneapolis, MN 55455, USA

**Keywords:** mother–infant bonding, postnatal care, Uganda, nursing, midwifery care

## Abstract

Impaired maternal–infant bonding can have a negative impact on the mother–infant relationship, affecting the social, emotional, and cognitive development of a child. In Uganda, there is a paucity of literature on impaired maternal–infant bonding. This quantitative, cross-sectional study aimed to determine the prevalence and factors associated with impaired maternal–infant bonding. Postnatal mothers (*n* = 422) attending the Young Child Clinic at Kawempe National Referral Hospital participated in the study. Maternal–infant bonding was measured using the Postpartum Bonding Questionnaire (PBQ). Participants with a score ≥ 13 on the PBQ were considered to have impaired maternal–infant bonding. The prevalence of impaired maternal–infant bonding among mothers was 45% (190/422). Logistic regression was used to determine factors associated with impaired maternal–infant bonding. Unmarried mothers (AOR = 2.05, 95% [CI = 1.03–4.09], *p* = 0.041), unplanned pregnancy (AOR = 5.19, 95% [CI = 3.07–8.82], *p* < 0.001), first-time mothers (AOR = 2.46, 95% [CI = 1.37–4.43], *p* = 0.003), female infant (AOR = 1.80, 95% [CI = 1.13–2.86], *p* = 0.013), mothers with no/low education levels (AOR = 2.29, 95% [CI = 1.05–4.50], *p* = 0.036), and those who delivered post term (AOR = 2.49, 95% [CI = 1.10–5.67], *p* = 0.028) were more likely to have impaired maternal–infant bonding. Nurses and midwives in postnatal care should include maternal–infant bonding within their client’s assessment and provide supportive mother-centered care. Interventions to improve maternal–infant bonding should be created and implemented in clinical practice.

## 1. Introduction

Mother–infant bonding was first described five decades ago as an emotional tie between a mother and baby [1]. Others have since described it as an affective connection or passionate tie between the mother and her infant [2,3]. Maternal–infant bonding is characterized by emotional warmth and affection between the mother and the infant [4]. It is considered to be the basis for the child’s later attachment and sense of self [4]. The development of this bond begins during intrauterine life and continues throughout the child’s life [5]. However, the first year of life has been identified as a vital time period for maternal–infant bonding [6].

A strong bond between mother and infant is important for the overall well-being and growth of the child, improving both physical and psychological development [6,7]. This bond plays a pivotal role in fostering social and emotional growth, as well as enhancing cognitive abilities in the child [8]. Moreover, nurturing positive maternal–infant relationships creates healthy interpersonal connections throughout the child’s life, facilitating smoother interactions with others and equipping them with essential parenting skills for the future [3]. Additionally, a robust maternal–newborn relationship significantly influences the mother’s mental well-being and her approach to parenting [8,9].

The disruption of mother–infant bonding affects the infant’s social, emotional, behavioral, and cognitive development [10]. Poor bonding is associated with complications including failure to thrive, psychosocial disorder, separation anxiety disorder, avoidant personality disorder, delinquency, and learning problems [10,11]. The prevalence of bonding problems among mother–infant dyads has been reported in studies conducted in the Global North and South and varies greatly from 4 to 45% [2,11,12,13,14,15].

The factors associated with impaired maternal–infant bonding among mothers from developing countries are not well documented. Given the impact of impaired postnatal maternal–infant bonding on the future health and development of the child, it is essential to examine the factors associated with impaired bonding in order to identify and support mothers who are at an increased risk of bonding problems. This study aims to determine the prevalence and factors associated with impaired maternal–infant bonding in the postnatal period.

## 2. Materials and Methods

### 2.1. Study Design and Setting

A quantitative descriptive, cross-sectional study was conducted to determine the prevalence and factors associated with impaired maternal–infant bonding among postnatal women and infants at the Young Child Clinic (YCC) at Kawempe National Referral Hospital. Kawempe Hospital is a national referral, clinical, training, and research hospital. The hospital has a 200-bed capacity and is located in Kawempe Division, one of the five administrative units of the Kampala Capital City Authority. The hospital is located approximately 5 km north of Mulago National Specialized Referral Hospital, along the Kampala–Gulu Highway. This location is approximately 8 km north of the central business district of the city. The hospital offers free services in obstetrics and gynecology, pediatrics, adolescent health, and HIV/AIDS care. The YCC is an outpatient clinic located within Kawempe National Referral Hospital which offers immunization services for infants, health education and referral services for postnatal mothers. The YCC operates from Monday to Friday with nurses and midwives, seeing over 200 mother–infant dyads weekly. 

### 2.2. Study Sample

The study population included all mothers who had given birth to live babies in the previous 14 weeks and had infants receiving care from the YCC. Participants were recruited as they brought their newborn to the YCC for immunizations as per the National immunization schedule. Mothers below 18 years old were considered emancipated minors and included in the study. The study excluded mothers whose babies required higher levels of pediatric care for medical conditions (e.g., failure to thrive, congenital conditions) and those who did not understand Luganda or English.

The study participants were consecutively recruited in the study, a sampling procedure in which every participant meeting the inclusion criteria is recruited until the required sample size is achieved [16]. Consecutive recruitment reduces selection bias, maintains high response rate, and ensures equitable access to recruitment opportunities for all eligible individuals [16]. Every mother who met the inclusion criteria was given an opportunity to participate in the study. The number of study respondents was obtained using Kish’s formula [17]. When accounting for a 10% non-response rate, 422 participants were required to complete the study.

### 2.3. Study Variables

The dependent variable examined was maternal–infant bonding, which was measured using the Postpartum Bonding Questionnaire (PBQ). The independent variables examined included sociodemographic factors such as a woman’s age, level of education, employment status, marital status, time spent with the baby, and social support; maternal factors included parity, still births, planned pregnancy, antenatal attendance, type of delivery, birth complications, and skin-to-skin contact; and infant factors included sex, birth weight, gestational age, condition at birth, current weight, age, illness, and breastfeeding status.

### 2.4. Data Collection Tools

Data were collected via a structured questionnaire with two sections. Section one contained sociodemographic, maternal, and infant factors. Section two included the PBQ, which is a 25-item survey designed to detect mothers at risk for bonding disorders [18,19]. The PBQ was specifically chosen to measure mother–infant bonding in this study because it is a commonly used measure that has been shown to be reliable and valid across different countries, languages, and cultures [11,14,19,20,21,22,23,24,25]. In Africa, it has been used in Egypt [15] and Ethiopia [2].

The PBQ has four subscales measuring quality of the maternal–infant bond, rejection and pathological anger towards the baby, infant-focused anxiety, and risk of abuse. The items are rated by the mother on a six-point scale. Each item is scored between 0 and 5; negatively phrased items are rescored. The scores from the 25 items are summed to produce the maternal–infant bonding score. Total scores range from 0 to 125, with a higher score indicating a bonding disorder [18]. In the current study, we used a cut-off score of 13 to categorize mothers as having impaired maternal–infant bonding. This cut-off was based on a previous study that used similar cut-offs [14].

### 2.5. Data Collection Procedure

The study was approved by the School of Health Science Research and Ethics Committee (MAKSHSREC-2022-280). Administrative approval was granted by Kawempe National Referral Hospital and the YCC in-charge. The researchers approached mothers at the YCC who had completed their immunization visits with their infants and explained the study’s purpose, risk, and benefits. Written informed consent was secured from mothers who were willing to participate in the study. After securing written informed consent, the research team member and participant moved to a private room in the clinic and the research team member administered the questionnaire, which took, on average, 40 min.

### 2.6. Data Analysis

The data were entered into Excel, cleaned, and analyzed using STATA version 16 software. There were no missing data. Descriptive statistics of sociodemographic, maternal, and infant characteristics were computed. Categorical variables were reported using frequency and proportions. Continuous variables were summarized using median, mean, and standard deviation.

The individual items on the PBQ were summed for each participant to compute the total maternal–infant bonding score. Descriptive statistics (range, mean, standard deviation) were obtained for all items and subscales of the PBQ. To assess the internal reliability of the PBQ, Cronbach’s alpha was computed. To determine the prevalence of impaired maternal–infant bonding, mothers with scores ≥ 13 on the PBQ were computed as a percentage of the total number of participants.

Logistic regression was used to determine association between the various independent variables and impaired maternal–infant bonding, using odds ratio (OR) and 95% confidence interval at bivariate and multivariate analysis. At the bivariate analysis level, factors with *p*-value ≤ 0.05 and plausible variables were included in the multivariate model. A *p*-value of ≤ 0.05 was considered significant for the final model.

## 3. Results

The study aimed to determine the prevalence and factors associated with impaired maternal–infant bonding among 422 mothers attending YCC at Kawempe Hospital.

### 3.1. Sociodemographic Characteristics

The mean age of respondents was 26.2 years. More than three-quarters were married or living with a partner, and slightly more than half had a secondary level of education. Of the 181 mothers who were employed, more than half reported taking their babies with them to work. Fifteen percent reported that they had no support with caring for the baby (Table 1).

### 3.2. Maternal Characteristics

The average number of pregnancies for participants was two. Approximately 60% (*n* = 255) reported the pregnancy as unplanned and 73% (*n* = 397) of participants reported attending the antenatal clinic between four and seven times during the pregnancy. The majority (*n* = 301) of births were spontaneous vaginal deliveries, 99.5% of all births occurred in a health facility, and just more than a third (*n* = 158) reported complications during delivery. A small number (*n* = 53) never put the babies on the chest after delivery (Table 2).

### 3.3. Infant Characteristics

The mean age of the babies was 8 weeks. Slightly less than a half (*n* = 196) of the respondents gave birth to male babies. The majority of infants were born at term (*n* = 363) and more than three-quarters (*n* = 358) indicated that they exclusively breastfed their babies. The average weight at birth was 3.06 kg (Table 3).

### 3.4. Maternal–Infant Bonding

Maternal–infant bonding was measured using the PBQ. The overall Cronbach’s alpha of the PBQ in this study was 0.81, indicating good internal consistency. The total participant scores ranged from 0 to 82, with a mean score of 12.47 and median of 12. Mothers with scores ≥ 13 were categorized as having impaired bonding. More than 4 in 10 (45%) mothers experienced impaired bonding. Descriptive statistics for the PBQ including subscales are noted in Table 4.

### 3.5. Association between Demographic Factors and Impaired Maternal–Infant Bonding among Mothers Attending the YCC at Kawempe Hospital

Bivariate analysis was completed to identify the sociodemographic factors associated with impaired maternal–infant bonding among mothers attending the YCC (Table 5). Young mothers (age 15–24) were more likely to report bonding problems compared with women aged 25–34 (OR = 1.61, 95% CI = 1.07–2.42, *p* = 0.021). Mothers who were not married or living with a partner were more likely to report concerns with bonding (OR = 2.96, 95% CI = 1.70–5.14, *p* < 0.001). Mothers with no formal education or primary school only experienced more bonding issues than those with tertiary education (OR = 3.07, 95% CI = 1.60–0.62, *p* = 0.001). Mothers who were not taking babies with them to work had about 4 times greater odds of bonding problems compared to those who were taking their babies with them to work (OR = 3.76, 95% CI = 2.01–7.05, *p* < 0.001) and those with no support had 81% greater odds of developing bonding problems compared to those who had support from their husbands and other people (OR = 1.81, 95% CI = 1.07–3.05, *p* = 0.027).

### 3.6. Association between Maternal Factors and Impaired Maternal Bonding among Mothers Attending the YCC

First-time mothers had greater odds of developing bonding problems compared to those who had delivered before (OR = 1.89, 95% [CI = 1.28–2.78], *p* = 0.001). Mothers reporting an unplanned pregnancy had 6 times greater odds of developing bonding problems compared to those who had planned for the pregnancy (OR = 6.16, 95% [CI = 3.92–9.70], *p* < 0.001). Surprisingly, those who delivered by caesarean section had decreased odds of developing bonding problems compared to those who had a spontaneous vaginal delivery (OR = 0.64, 95% [CI = 0.413–0.98], *p* = 0.041) and mothers who had complications during delivery also had lower odds of developing bonding problems compared to those who did not have any complications (OR = 0.66, 95% [CI = 0.44–0.98], *p* = 0.041). Table 6 describes the maternal factors associated with impaired maternal–infant bonding.

### 3.7. Association between Infant Factors and Impaired Maternal Bonding among Mothers Attending the YCC

Mothers who delivered female infants had a greater likelihood of poor maternal–infant bonding compared to those who delivered male infants (OR = 1.73, 95% [CI = 1.19–2.57], *p* = 0.005). Mothers who gave birth to post term newborns had 2 times greater odds of bonding problems compared to those who gave birth to term babies (OR = 2.07, 95% [CI = 1.01–4.27], *p* = 0.048). Exclusive breastfeeding or breastfeeding within the first hour was not significantly associated with maternal–infant bonding. Table 7 describes the association between infant factors and maternal–infant bonding.

### 3.8. Multivariate Analysis of Independent Variables and Impaired Maternal–Infant Bonding

A logistic regression model was developed to assess the factors associated with impaired maternal–infant bonding for the variables that had a *p* ≤ 0.05. Age, marital status, level of education, baby support, planned pregnancy, parity, mode of delivery, birth complications, skin-to-skin contact, sex of the baby, and gestational age of the baby were included in multivariate analysis. This model explained 20% of variability (adjusted r^2^ = 0.20) in maternal–infant bonding.

Table 8 describes the findings of the multivariate analysis. Unmarried mothers had 2 times greater odds of developing bonding problems compared to those who were married/living with a partner (AOR = 2.05, 95% [CI = 1.03–4.09], *p* = 0.041); those with unplanned pregnancy had 5 times greater odds of developing bonding problems (AOR = 5.19, 95% [CI = 3.07–8.82], *p* < 0.001). The odds of developing infant bonding problems were more than two times higher among first-time mothers compared to mothers who had had two or more pregnancies (AOR = 2.46, 95% [CI = 1.37–4.43], *p* = 0.003). Mothers with no education or primary level only were more likely to develop maternal–infant bonding issues (AOR = 2.29, 95% [CI = 1.05–4.50], *p* = 0.036). Mothers who delivered female babies had greater odds of developing bonding problems compared to those who delivered males (AOR = 1.80, 95% [CI = 1.13–2.86], *p* = 0.013) and those who delivered post term were more likely to experience bonding issues compared to those who delivered at term (AOR = 2.49, 95% [CI = 1.10–5.67], *p* = 0.028).

## 4. Discussion

Impaired maternal–infant bonding has implications on the health and well-being of the mother and infant. This study determined the prevalence and factors associated with impaired maternal–infant bonding in a sample of 422 women in Uganda. The prevalence of impaired maternal–infant bonding was 45% and was more likely in first-time mothers, those with low or no education, mothers with an unplanned pregnancy, unmarried mothers, those who gave birth to a female infant, and those who delivered post term.

International studies have found a wide range in the prevalence of maternal–infant bonding disorders [2,11,12,13,14,15]. One study in Egypt found 4.2% of mothers experienced impaired bonding, which is significantly lower than this study [15]. Another study completed in India found bonding disorders in 24% of healthy postpartum mothers, yet found a similar rate of 45% in mothers with a psychiatric disorder [26]. A study in the United States found a comparatively lower rate (23%) of parent–infant bonding disorder in parents with post-traumatic stress disorder and depression [27].

The variation in prevalence of impaired maternal–infant bonding in the postpartum period could be due to the different measurement tools used to assess maternal–infant bonding disorders [28,29,30]. It may also be attributed to the different cut-off scores used across studies [14,18,26]. Finally, a diversity in setting, language, or culture could lead to differences [11,20,26].

The low-resource context of Uganda may have implications for impaired maternal–infant bonding. In Uganda, there are no established processes for screening mothers for bonding disorders with their babies. The current study identifies a high prevalence of maternal–infant bonding disorders, which could cause long-term risks to the mother and child including impaired mental and cognitive development of the child. Hence, there is a need to integrate maternal–infant bonding assessment into routine antenatal and postnatal care.

Mothers with high social or economic strain may have a decreased ability to establish strong maternal–infant bonds. This strain may be exacerbated if the mother does not have social support. In this study, not being married or living with a partner was associated with impaired maternal–infant bonding. This may be due to single mothers having a larger burden of childrearing or societal stigma, impacting the bonding process. In Uganda, there is a taboo against sex outside of marriage. Furthermore, mothers might not have planned for the pregnancy or lacked support from the family to meet the costs of raising a child, which may impact attachment [31,32,33]. The findings are consistent with Figueiredo et al.’s study which found unmarried mothers had a worse emotional connection with their infants [34] and Joas and Mohler who found married women had improved maternal–infant bonding [35]. However, it contradicts findings from Lehnig et al. which noted that married women had a higher risk of developing bonding problems [36]. Two studies performed in Turkey have found no significant association between marital status and impaired maternal–infant bonding [7,37].

The study findings revealed that level of education was significantly associated with maternal–infant bonding. Mothers with primary or no education were 2 times more likely to develop bonding problems compared to those with higher levels of education. This is in agreement with studies carried out in Ethiopia that revealed that mothers with low or no education had poor bonding towards their babies [2]. The findings contradict other studies that showed that low education was significantly related to positive postnatal bonding [6,36,38,39]. Other studies have not shown a significant difference between level of education and impaired maternal infant bonding [7].

In this study, age had no significant association with impaired maternal–infant bonding. This does not reflect findings from two studies that found that young mothers may be more likely to have impaired bonding with their infant [37,40]. Conversely, other studies found that impaired maternal–infant bond was higher in older mothers [7,39]. In this study, employment status had no significant association with impaired maternal–infant bonding; this is contrary to studies conducted in Ethiopia and Saudi Arabia that showed mothers who were unemployed had poor bonding with their babies [2,41].

In this study, maternal factors such as parity and whether or not the pregnancy was planned had a significant association with impaired maternal–infant bonding. The results show that women who had unplanned pregnancy had almost 6 times greater odds of developing bonding problems with their babies than those who had planned to get pregnant. Nakano et al. note that unplanned pregnancy may create negative feelings towards the fetus during pregnancy which may extend to after birth [13]. This agrees with Rossen et al.’s work that found unplanned pregnancy was associated with impaired maternal–infant bonding [31]. On the other hand, women with planned pregnancies have more feelings of closeness and tenderness toward their fetuses in pregnancy; this has been reported to be predictor of higher bonding in the postpartum period [31,42,43]. The findings of this study are contrary to other studies that found no significant relationship between planned or unplanned pregnancy and maternal–infant bonding [15,44,45,46]. Tichelman et al. argued that there was no significant association between planned pregnancy and maternal–infant bonding and, thus, having an unplanned pregnancy would not prevent a mother from developing positive feelings towards her child [45].

Findings in this study indicate that first-time mothers were two times more likely to develop impaired bonding compared to mothers who had delivered two or more times. The transition to motherhood can be a stressful time given the new demands and responsibilities of parenting [24]. First-time mothers may be anxious and feel less confident about taking care of their babies [13,32]. This is in agreement with other studies that showed higher bonding impairment in first-time mothers than mothers who already had other children [13,24,44]. Similar to other studies, other maternal factors like mode of delivery, antenatal attendance, birth complications, and skin-to-skin contact had no significant effect on maternal–infant bonding [2,45,47]. However, it should be noted that two previous studies showed that skin-to-skin contact between mother and baby enhances maternal–infant bonding [6,47].

The current results showed that there was a statistically significant relationship between impaired bonding and the sex of the baby. Mothers who gave birth to female infants had almost 2 times greater odds of developing bonding problems compared to those who gave birth to males. This agrees with a study carried out in Egypt where poor bonding was more prevalent in mothers who had females [15]. Conversely, a study performed in Saudi Arabia showed that poor bonding was more common in mothers who had male infants [44]; Daglar and Nur found no significant association between sex and maternal–infant bonding [7]. Our study indicates that mothers may have a preference for the sex of the child. This is consistent with findings from one study that noted there is a strong cultural preference in Uganda for male children [48]. It would be important for nurses and midwives to query mothers postnatally about their feelings about the sex of their infant. A mother who delivers a baby of the non-preferred sex should be offered counseling and support in developing bonds with their baby.

Findings in this study show that mothers who delivered post term babies were twice as likely to develop bonding problem compared to mothers who had term babies. Other infant factors like weight and feeding type were not significantly associated with impaired maternal–infant bonding in this study. This is consistent with other studies [40,44,49,50]. Conversely, a study completed in Ethiopia found a significant relationship between not breastfeeding and impaired maternal–infant bonding [2]. The postnatal period is a sensitive time and can impact the emotional bond between mothers and their infant [51]. Therefore, mothers should be assessed for bonding problems and supported in forming a good and healthy bond with their babies.

### Strengths and Limitations

The strengths of this study include using the PBQ, a valid and reliable tool which showed an acceptable internal consistency in this study. The tool was available in two languages to allow for data collection from different patient demographics. The large sample size provided stronger and more reliable results with a small margin of error. The study will provide a baseline knowledge in informing policy in Uganda and for future studies.

Limitations of this study include the study design. The cross-sectional design does not allow participants to be followed up to determine if bonding scores changed over time. Additionally, participants may have felt pressure to answer based on social desirability. This bias was reduced by ensuring the anonymity and confidentiality of participants. Finally, the study was conducted in a large urban hospital; hence, generalizability may be limited.

## 5. Conclusions

Nurses and midwives in antenatal and postnatal care should include maternal–infant bonding within their client’s assessment and provide supportive mother-centered care. Nurses and midwives are encouraged to promote individual factors that enhance maternal–infant bonding and also give timely management to mothers found to be at risk for impaired maternal–infant bonding. Guidelines and interventions to improve maternal–infant bonding should be created and implemented in clinical practice. This will require additional research and investment in understanding intervention best practices. However, given the findings of this study, screening at-risk mothers for impaired bonding could be a way to identify and provide support to new parents.

The findings of this study highlight the importance of maternal–infant bonding. Incorporating the routine assessment of maternal–infant bonding during the antenatal and postnatal period may enable early identification of mothers at risk of bonding problems. Healthcare workers should be trained on proper assessment and treatment to ensure timely management to support the mother–infant bond.

Replication of the same study in different regions of Uganda, both in rural and urban areas, to compare and validate the present study findings is recommended. Future research should consider the association between postnatal depression and anxiety and maternal–infant bonding, and longitudinal studies to assess the long-term impact of impaired maternal–infant bonding.

## Figures and Tables

**Table 1:** Sociodemographic characteristics.

Variable	Frequency (*n*)	Percent (%)	Mean (± SD)
**Age**			
15–24	179	42.4	26.2 (± 5.57)
25–34	202	47.9	
35 and above	41	9.7	
**Marital status**			
Never married	43	10.2	
Married/Living together	355	84.1	
Separated or divorced	24	5.7	
**Level of education**			
No education	17	4.0	
Primary	115	27.3	
Secondary	230	54.5	
Tertiary	60	14.2	
**Mother’s employment status**			
Employed	181	42.9	
Not employed	241	57.1	
**Type of employment**			
No employment	241	57.1	
Self-employed or business	127	30.1	
Civil servant	54	12.8	
**Takes baby with her to workplace**			
Yes	107	59.1	
No	74	40.9	
**Maternal support for baby care**			
None	65	15.4	
Husband or partner	282	66.8	
Others	75	17.8	

**Table 2:** Maternal characteristics.

Variable	Frequency (*n*)	Percent (%)	Mean (SD)
**Parity**			2.07 (± 1.32)
1	210	49.8	
≥2	212	50.2	
**Ever lost a child**			
Yes	61	14.5	
No	361	85.5	
**Intended** **pregnancy**			
Yes	167	39.6	
No	255	60.4	
**Antenatal clinic visits**			5.75 (± 1.65)
<4 visits	25	5.9	
4–7 visits	397	73.0	
≥8 visits	89	21.1	
**Place of delivery**			
Home	2	0.5	
Public Hospital	381	90.3	
Private Hospital	39	9.2	
**Mode of delivery**			
Vaginal	301	71.3	
Caesarean	121	28.7	
**Pregnancy complication**			
None	264	62.6	
Complication	158	37.4	
**Skin-to-skin contact**			
Immediately	284	67.3	
First 24 h	85	20.1	
Never	53	12.6	

**Table 3:** Infant characteristics.

Variable	Frequency (*n*)	Percentage (%)	Mean (SD)
**Age of baby in weeks**	422	100	8.86 (± 3.47)
**Sex of baby**			
Female	226	53.6	
Male	196	46.2	
**Preferred sex**			
Female	356	84.4	
Male	66	15.6	
**Condition of baby at birth**			
Preterm	25	5.9	
Full term	363	86.0	
Post term	34	8.1	
**Weight at birth in kg**			3.07 (± 0.54)
Below 2.5	37	8.8	
2.5–3.5	293	69.4	
Above 3.5	92	21.8	
**Baby cried immediately at birth**			
Yes	400	94.8	
No	22	5.2	
**Baby taken to NICU after birth**			
Yes	49	1.6	
No	373	88.4	
**Baby hospitalized since discharge**			
Yes	8	1.9	
No	414	98.1	
**Method of feeding baby after birth**			
Exclusively breast fed	358	84.8	
Both	64	15.2	
**Baby breast fed in the first hour after birth**			
Yes	291	69.0	
No	131	31.0	
**Sleep with baby in the same bed**			
Yes	416	98.6	
No	6	1.4	

**Table 4:** Postpartum Bonding Questionnaire results.

Postpartum Bonding Questionnaire	Number of Items	Mean	Standard Deviation	Median	Range
Subscale 1 (quality of maternal bond)	12	6.86	5.1	07	0–46
Subscale 2 (rejection and anger)	07	2.36	3.30	02	0–28
Subscale 3 (infant-focused anxiety)	04	3.24	1.84	03	0–12
Subscale 4 (incipient abuse)	02	0.01	0.84	00	0–01
**Total Item Score**	25	12.47	9.03	12	0–82

**Table 5:** Association between sociodemographic factors and maternal–infant impaired bonding.

Variable	Normal Bonding	Impaired Bonding	Odds Ratio (95% CI)	*p-*Value
**Age**				
25–34	122 (60.4)	80 (39.6)	Ref	
15–24	87 (48.6)	92 (51.4)	1.61 (1.07–2.42)	**0.021**
35 and above	23 (56.1)	18 (43.9)	1.19 (0.61–2.35)	0.609
**Marital status**				
Married/living together	210 (59.2)	145 (40.8)	Ref	
Not married	22 (32.8)	45 (67.2)	2.96 (1.70–5.14)	**<0.001**
**Education level**				
Tertiary	42 (70.0)	18 (30.0)	Ref	
Secondary	133 (57.8)	97 (42.2)	1.70 (0.92–5.89)	0.088
No education/primary	57 (43.2)	75 (56.8)	3.07 (1.60–0.62)	**0.001**
**Job status**				
Yes	107 (59.1)	74 (40.9)	Ref	
No	125 (51.9)	116 (47.1)	1.34 (0.91–1.98)	0.139
**Take baby to work (*n*= 181)**				
Yes	77 (72.0)	30 (28.0)	Ref	
No	30 (40.5)	44 (59.5)	3.76 (2.01–7.05)	**<0.001**
**Baby support**				
Husband/other	203 (57.3)	151 (42.6)	Ref	
None	29 (42.6)	39 (57.4)	1.81 (1.07–3.05)	**0.027**

Bold: statistically significant with *p* < 0.05

**Table 6:** Association between maternal factors and impaired maternal–infant bonding.

Variable	Normal Bonding	Impaired Bonding	Odds Ratio (95% CI)	*p*-Value
**Parity**				
≥2	133 (62.7)	79 (37.3)	Ref	
1	99 (47.1)	111 (52.9)	1.89 (1.28–2.78)	**0.001**
**Lost a child**				
No	199 (55.1)	162 (44.9)	Ref	
Yes	33 (54.1)	28 (45.9)	1.04 (0.60–1.80)	0.882
**Planned pregnancy**				
Yes	133 (79.6)	34 (20.4)	Ref	
No	99 (38.8)	156 (61.2)	6.16 (3.92–9.70)	**<0.001**
**Antenatal visits**				
≥4	214 (53.9)	183 (46.1)	Ref	
<4	18 (72.0)	7 (38.0)	0.54 (0.19–1.11)	0.084
**Mode of delivery**				
Vaginal	156 (51.8)	145 (49.1)	Ref	
Caesarean	76 (62.8)	45 (37.2)	0.64 (0.41–0.98)	**0.041**
**Complications**				
None	135 (51.1)	129 (48.9)	Ref	
Complication	97 (61.2)	61 (38.8)	0.66 (0.44–0.98)	**0.041**
**Carried baby on chest**				
Immediately	145 (51.1)	139 (49.9)	Ref	
First 24 h	45 (52.9)	40 (47.1)	0.93 (0.57–1.51)	0.760
Never	42 (79.3)	11 (20.7)	0.27 (0.14–0.55)	**<0.001**

Bold: statistically significant with *p* < 0.05.

**Table 7:** Association between infant factors and impaired maternal–infant bonding.

Variable	Normal Bonding	Impaired Bonding	Odds Ratio (95% CI)	*p*-Value
**Age of the baby**				
≤8 weeks	115 (50.9)	111 (49.1)	Ref	
>8 weeks	117 (59.6)	79 (40.4)	0.70 (0.46–1.03)	0.070
**Sex of the baby**				
Male	122 (62.2)	72 (37.8)	Ref	
Female	110 (48.7)	116 (51.3)	1.74 (1.18–2.57)	**0.005**
**Sex preferred**				
Yes	201 (56.5)	155 (43.5)	Ref	
No	31 (47.0)	35 (53.0)	1.46 (0.86–2.48)	0.156
**Condition of the baby**				
Full term	204 (56.2)	159 (43.8)	Ref	
Preterm	15 (60.0)	10 (40.0)	0.86 (0.37–1.95)	0.711
Post term	13 (38.2)	21 (61.8)	2.07 (1.01–4.27)	**0.048**
**Baby weight at birth**				
<2.5 kg	23 (62.2)	14 (37.8)	Ref	
2.5–3.5 kg	146 (49.8)	147 (50.2)	1.65 (0.82–3.34)	0.160
>3.5 kg	63 (68.5)	29 (36.5)	0.76 (0.34–1.68)	0.492
**Baby cried at birth**				
Yes	214 (54.5)	182 (45.5)	Ref	
No	14 (63.6)	8 (36.4)	0.68 (0.28–1.67)	0.404
**Baby taken to ICU**				
Yes	30 (61.2)	19 (38.8)	Ref	
No	202 (54.2)	171 (45.8)	1.33 (0.73–2.46)	0.351
**Forms of feeding**				
Exclusive breastfeeding	190 (53.1)	168 (46.9)	Ref	
Bottle/breastfeeding	42 (65.6)	22 (34.4)	0.59 (0.34–1.03)	0.065
**Breastfed within first hour**				
Yes	153 (52.2)	139 (47.8)	Ref	
No	80 (61.1)	51 (38.9)	0.70 (0.46–1.06)	0.092

Bold: statistically significant with *p* < 0.05.

**Table 8:** Factors associated with impaired maternal–infant bonding.

Variable	Normal Bonding	Impaired Bonding	Odds Ratio (95% CI)	*p-*Value	Adjusted Odds Ratio (95% CI)	*p-*Value
**Age**						
25–34	122 (60.4)	80 (39.6)	Ref		Ref	
15–24	87 (48.6)	92 (51.4)	1.61 (1.07–2.42)	0.021	0.92 (0.50–1.60)	0.693
35 and above	23 (56.1)	18 (43.9)	1.19 (0.61–2.35)			
**Marital status**						
Married/living together	210 (59.2)	145 (40.8)	Ref		Ref	
Not married	22 (32.8)	45 (67.2)	2.96 (1.70–5.14)	<0.001	2.36 (1.20–4.61)	**0.021**
**Education level**						
Tertiary	42 (70.0)	18 (30.0)	Ref		Ref	
Secondary	133 (57.8)	97 (42.2)	1.70 (0.92–3.14)	0.088		
None/primary	57 (43.2)	75 (56.8)	3.07 (1.60–5.89)	0.001	2.29 (1.05–4.50)	**0.036**
**Baby support**						
Husband/other	203 (57.3)	151 (42.6)	Ref		Ref	
None	29 (42.6)	39 (57.4)	1.81 (1.07–3.05)	0.027	1.02 (0.55–1.91)	0.110
**Parity**						
≥2	79 (70.0)	39 (30.0)	Ref		Ref	
1	153 (50.3)	151 (49.7)	1.89 (1.28–2.78)	0.001	2.49 (1.40–4.42)	**0.002**
**Planned pregnancy**						
Yes	133 (79.6)	34 (20.4)	Ref		Ref	
No	99 (38.8)	156 (61.2)	6.16 (3.92–9.70)	<0.001	5.88 (3.52–9.81)	**<0.001**
**Mode of delivery**						
Vaginal	156 (51.8)	145 (49.1)	Ref		Ref	
Caesarean	76 (62.8)	45 (37.2)	0.64 (0.41–30.98)	0.041	1.01 (0.32–3.25)	0.983
**Complications**						
None	135 (51.1)	129 (48.9)	Ref		Ref	
Complication	97 (61.2)	61 (38.8)	0.66 (0.44–0.98)	0.041	0.63 (0.34–1.15)	0.132
**Skin-to-skin contact**						
Immediately	145 (51.1)	139 (49.9)	Ref		Ref	
First 24 h	45 (52.9)	40 (47.1)	0.93 (0.57–1.51)			
Never	42 (79.3)	11 (20.7)	0.27 (0.14–0.55)	<0.001	0.81 (0.25–2.68)	0.733
**Baby’s sex**						
Male	122 (62.2)	72 (37.8)	Ref		Ref	
Female	110 (48.7)	116 (51.3)	1.74 (1.18–2.57)	0.005	1.82 (1.15–2.87)	**0.010**
**Gestational age**						
Full term	204 (56.2)	159 (43.8)	Ref		Ref	
Preterm	15 (60.0)	10 (40.0)	0.86 (0.37–1.95)			
Post term	13 (38.2)	21 (61.8)	2.07 (1.00–4.27)	0.048	2.49 (1.10–5.67)	**0.028**

Bold: statistically significant with *p* < 0.05.

## Data Availability

The data are available upon request to the corresponding author.
